# An Enriched Environment Enhances Angiogenesis Surrounding the Cingulum in Ischaemic Stroke Rats

**DOI:** 10.1155/2020/8840319

**Published:** 2020-11-12

**Authors:** Xueyan Shen, Lu Luo, Fei Wang, Kewei Yu, Hongyu Xie, Shan Tian, Gang Liu, Chunrong Bao, Yunhui Fan, Ying Xing, Nianhong Wang, Siyue Li, Li Liu, Qun Zhang, Yi Wu

**Affiliations:** ^1^Department of Rehabilitation Medicine, Huashan Hospital, Fudan University, Shanghai 200041, China; ^2^Department of Hand Surgery, Huashan Hospital, Fudan University, Shanghai 200040, China; ^3^Department of Hand and Upper Extremity Surgery, Jing'an District Central Hospital of Shanghai, Fudan University, Shanghai 200040, China; ^4^School of Rehabilitation Science, Shanghai University of Traditional Chinese Medicine, Shanghai 201203, China

## Abstract

An enriched environment (EE) has been demonstrated to improve functional recovery in animal models of ischaemic stroke through enhancing vascular endothelial growth factor- (VEGF-) mediated neuroprotection accompanied by angiogenesis in the ischaemic hemisphere. Whether EEs also promote VEGF-mediated neuroprotection and angiogenesis in the contralateral hemisphere remains unclear. Here, we explored the effect of EEs on VEGF expression and angiogenesis within the contralateral cerebral cortex in a rat middle cerebral artery occlusion/reperfusion (MCAO/r) model. We assessed the expression levels of platelet endothelial cell adhesion molecule-1 (CD31), VEGF, and endothelial nitric oxide synthase (eNOS) in the whole contralateral cerebral cortex using Western blotting assay but did not find an increase in the expression of CD31, VEGF, or eNOS in MCAO/r rats housed in EEs, which suggested that EEs did not enhance the overall expression of VEGF and eNOS or angiogenesis in the entire contralateral cortex. We further analysed the local effect of EEs by immunohistochemistry and found that in and around the bilateral cingulum in MCAO/r rats housed in EEs, haematopoietic progenitor cell antigen- (CD34-) positive endothelial progenitor cells were significantly increased compared with those of rats housed in standard cages (SCs). Further experiments showed that EEs increased neuronal VEGF expression surrounding the cingulum in MCAO/r rats and robustly upregulated eNOS expression. These results revealed that EEs enhanced angiogenesis, VEGF expression, and activation of the VEGF-eNOS pathway in and/or around the cingulum in MCAO/r rats, which were involved in the functional recovery of MCAO/r rats.

## 1. Introduction

After unilateral cerebral injury due to ischaemic factors, the cerebral hemisphere of the healthy side can compensatively control the affected limb through a potential neural pathway [1]. This functional compensation of the healthy side is an important way to restore limb function after unilateral cerebral ischaemia.

An enriched environment is a combination of stimuli, including a broad range of activities, increased numbers of social partners, and enriched objects and tactile stimuli [2]. EEs improve symptoms in animal models of stroke, neurodegenerative diseases, and mental diseases [3–6]. Different from the simple combination of single-stimulus interventions, EEs have specific effects on the nervous system in animals [7]. Increasing evidence shows that EEs have neuroprotective benefits in MCAO animal models [8, 9].

In the study of angiogenesis, VEGF is the most widely studied vascular growth factor [10, 11]. As a factor in one of the key pathways associated with angiogenesis, VEGF is mainly distributed in vascular endothelial cells and signals through vascular endothelial growth factor receptor-2 (VEGFR2), and the VEGF-mediated signalling pathway promotes the expression of eNOS [12]. VEGF can not only promote angiogenesis but also exert neuroprotective effects [13, 14]. The neurovascular unit (NVU) mechanism of cerebral ischaemic injury has been proposed in modern medicine in recent years [15]. Studying the distribution of vascular growth factors in the contralateral hemisphere in a unilateral stroke model can more intuitively show the interactions between angiogenesis and nerve reconstruction [16–18]. In a previous study, EEs increased CD31 levels and the expression levels of VEGF and its receptor after ischaemia-reperfusion injury in the ischaemic boundary zone [19, 20].

In our study, we proposed that EEs could also increase contralateral cortical angiogenesis through the VEGF pathway. We investigated the effects of EEs on CD31, VEGF, and eNOS protein expression in the contralateral cortical tissue of middle cerebral artery occlusion/reperfusion (MCAO/r) adult rats using Western blotting assay. For Western blotting assay, we collected the whole contralateral cerebral cortex, homogenised the tissue to extract the proteins, and discarded the subcortical part. Therefore, it is possible that the total amount of cortical protein remained unchanged, but the local distribution changed. Western blotting assay also could not reflect the protein changes in the subcortical structure. We tried to assess angiogenesis in subcortical structures or local areas of the contralateral cerebral cortex. We investigated the effects of EEs on the protein expression of CD34, a marker of endothelial progenitor cells, in middle cerebral artery occlusion/reperfusion adult rats using immunohistochemistry. We found that the protein expression of CD34 in and surrounding the bilateral cingulate was significantly different between the two groups. Then, we investigated the effects of EEs on VEGF, VEGFR2, eNOS, and hypoxia-inducible factor-1 alpha (HIF-1*α*) protein expression in the bilateral cingulum and border cortex of middle cerebral artery occlusion/reperfusion adult rats using immunohistochemistry. Our results suggested that EEs enhanced angiogenesis, VEGF expression, and activation of the VEGF-eNOS pathway in and/or around the cingulum of MCAO/r rats.

## 2. Materials and Methods

### 2.1. Animals

Healthy male Sprague-Dawley rats (weighing 250–280 g, 10 weeks old) were obtained from the Animal Holding Centre of the School of Pharmacy, Fudan University, and housed in specific pathogen-free environments with a room temperature of 22 ± 2°C and humidity of 50-60% under a 12 h light-dark cycle (6:00 a.m.-6:00 p.m.) with food and water available ad libitum. All experiments and procedures were performed according to the National Institutes of Health Guide for the Care and Use of Laboratory Animals (National Research Council, 1996) and approved by the Institutional Care of Experimental Animals Committee of Fudan University to minimise the suffering of animals (Shanghai, China; permit number: 20160858A232).

### 2.2. MCAO/r Surgery

MCAO/r surgery was performed according to a previously reported method. Briefly, the rats were anaesthetized with 2% pentobarbital sodium (40 mg/kg, i.p.), and then the left common carotid artery (CCA), external carotid artery (ECA), and internal carotid artery (ICA) were exposed. The ECA was ligated and dissected, and a round-tip nylon thread (2636, Beijing Cinontech Co., Ltd., China) was gently inserted (19 ± 1mm from the bifurcation of the left CCA) to block blood flow at the middle cerebral artery (MCA). The nylon thread was removed after 90 min. Each rat was neurologically evaluated with the Rogers scaling system 24 h after MCAO/r surgery. The Rogers scale was used to score functional status as follows: (0) no deficit; (1) failure to extend left forepaw; (2) pulling the tail decreases the grip of the left forelimb; (3) spontaneous movement in all directions, contralateral circling when pulled; (4) walking to the left or circling; (5) movement only when stimulated; (6) unresponsive to stimulation; and (7) dead [21]. Rats with a score between 3 and 4 were used for further experiments.

### 2.3. Environmental Intervention Protocols

The rats were randomly divided into standard cage (SC) and enriched environment (EE) groups according to the Rogers score 24 h after MCAO/r surgery.

#### 2.3.1. Standard Cage Group

The rats were housed individually in ventilated cages (cage size: 42cm × 37cm × 22cm) with 4 rats in each cage.

#### 2.3.2. Enriched Environment Group

**Figure 1:**
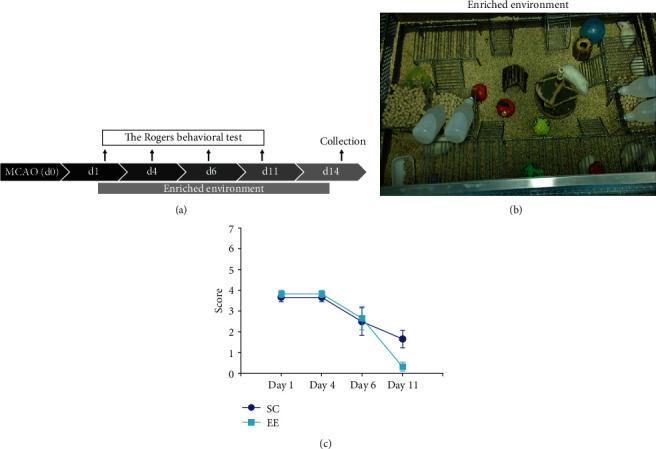
The enriched environment (EE) ameliorated the Rogers scores of MCAO rats. (a) A diagram showing the behavioural assay by the Rogers score on days 1, 4, 6, and 11 and the effect of the enriched environment for 14 days after MCAO/r. (b) Twelve rats with free access to food and water were housed in an enriched environment cage (120cm × 80cm × 100cm), in which a variety of toys were arranged for enriching the environmental stimulation. (c) A behavioural test using the Rogers score was performed on days 1, 4, 6, and 11 after MCAO/r. Repeated-measures analysis of variance (ANOVA) followed by Fisher's least significant difference (LSD) post hoc test was used for comparative analysis. *n* = 12 rats per group; ^∗^*P* < 0.05.

We designed enriched environmental cages (cage size: 120cm × 80cm × 100cm) (Figure 1(b)) according to the international standard and obtained the patent (Patent No.: ZL 201020560281.3). A variety of toys were arranged in the cage for environmental stimulation, including platforms, small wooden ladders, baffles, inclined boards, swings, and ropes for the rats to climb and play; cassettes, tunnels, and small animal statues of different colours and balls with different diameters for the rats to explore; various acoustic luminescent objects, odourous objects, and building blocks of different colours and shapes for multisensory stimulation; running wheels for the rats to exercise autonomously; and edible foam for the rats to bite. There were 12 rats housed in each cage. The environmental arrangement was changed every 7 days to maintain the novelty of the environmental stimulation.

### 2.4. Western Blotting Assay

Contralateral cortical tissue was collected 14 days after MCAO/r. The whole contralateral cerebral cortex was used for protein extraction, and the subcortical part was discarded. Whole protein extraction of the tissue samples was performed with RIPA lysis buffer (Beyotime, China) containing a protease inhibitor (Beyotime, China). Equal amounts of total protein samples were separated by sodium dodecyl sulfate-polyacrylamide gel electrophoresis (SDS-PAGE) and then electrotransferred to polyvinylidene difluoride (PVDF) membranes (Merck Millipore, Germany). The membranes were blocked with 5% bovine serum albumin for 1 h and incubated with goat anti-CD31 IgG (1 : 1000, Santa Cruz Biotechnology, USA), rabbit anti-VEGF IgG (1 : 1000, Abcam, USA), mouse anti-eNOS IgG (1 : 3000, Sigma-Aldrich, USA), and mouse anti-GAPDH IgG (1 : 5000, Santa Cruz Biotechnology, USA) primary antibodies at 4°C overnight. After being washed with TBST, the PVDF membranes were incubated for 2 h at room temperature with the corresponding horseradish peroxidase- (HRP-) conjugated antibodies (1 : 5000, KPL, USA). The chemiluminescent signals were scanned by a ChemiDoc MP Imaging System with Image Lab Software (Bio-Rad, USA) and analysed with a Gel-Pro Analyser (Media Cybernetics Inc., Silver Spring, MD, USA).

### 2.5. Immunofluorescence

Fourteen days after MCAO/r, the rats were deeply anaesthetized and transcardially perfused using 150 ml of precooled 0.01 M phosphate-buffered saline (PBS) followed by 200 ml of 4% paraformaldehyde (PFA). The brains were removed and postfixed in 4% PFA for 24 h at 4°C. The tissue was dehydrated with 10%, 20%, and 30% sucrose. Coronal sections (30 *μ*m) were prepared with a Leica CM1950 cryostat. For immunofluorescence analysis, the sections were blocked with 5% serum containing 0.3% Triton X-100 in 0.01 M PBS, followed by incubation with primary antibodies at 4°C overnight. All primary antibodies, including goat anti-CD34 IgG (1 : 100, Santa Cruz, USA), rabbit anti-VEGF IgG (1 : 300, Abcam, USA), rabbit anti-VEGFR2 IgG (1 : 300, Abcam, USA), and mouse anti-eNOS IgG (1 : 500, Sigma-Aldrich, USA), were diluted with 5% serum. After being washed with 0.01 M PBS, the sections were further incubated with goat anti-rabbit IgG H&L (Alexa Fluor® 488) (1 : 400, Abcam, USA) or goat anti-mouse IgG H&L (Alexa Fluor® 594) (1 : 400, Abcam, USA) secondary antibodies for 2 h at room temperature. After being thoroughly washed with PBS, the sections were mounted with antifade mounting medium (Southern Biotech, USA). Images were captured digitally using a CCD camera on an Olympus BX63 microscope connected to a computer. Images surrounding the cingulum in 9-12 sections per animal were acquired with 20x and 10x objectives and assessed by NIH ImageJ software.

### 2.6. Statistical Analysis

SPSS statistical (version 22) software (IBM, USA) and NIH ImageJ 14.0 software were used for statistical analysis. The data are presented as the means ± standarddeviations (SD). Student's *t*-tests or Kruskal-Wallis nonparametric tests of independent samples and repeated-measures ANOVA were used for statistical analysis. A value of *P* < 0.05 was considered significant.

## 3. Results

### 3.1. Enriched Environments Improved Movement Recovery in MCAO/r Rats

To establish a rat ischaemic stroke model, male Sprague-Dawley rats (250–280 g) underwent left middle cerebral artery occlusion/reperfusion (MCAO/r). Movement impairments in the rat MCAO/r model were examined using the Rogers scores 24 h after MCAO/r, and the MCAO/r rats with scores of 2-5 were randomly grouped into the enriched environment (*n* = 12 rats) and standard cage (*n* = 12 rats) groups, in which rats were housed in EEs and SCs for 14 days, respectively (Figures 1(a) and 1(b)). The Rogers scaling system was used to evaluate movement recovery in the MCAO/r rats on days 1, 4, 6, and 11 after MCAO/r (Figure 1(a)). Compared with that of the SC group, the Rogers score in the EE group was decreased on day 11 after MCAO/r; the SC group score was 1.67 ± 1.03, and the EE group score was 0.33 ± 0.52 (*F*_(1, 11)_ = 4.692, *P* = 0.037, *n* = 12, repeated-measures analysis of variance (ANOVA) followed by Fisher's least significant difference (LSD) post hoc test) (Figure 1(c)), which confirmed that, as reported in previous studies, the enriched environment improved movement recovery in the rat MCAO/r model.

### 3.2. Enriched Environments Did Not Increase Angiogenesis in the Whole Contralateral Cerebral Cortex

**Figure 2:**
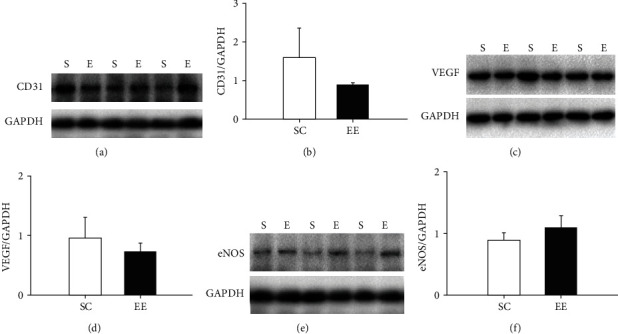
The effect of the enriched environment on contralateral cerebral cortical angiogenesis. (a) After 14 days of enriched environment exposure, the levels of CD31 within the contralateral cerebral cortex of MCAO rats in the standard cage (SC) and enriched environment (EE) groups were measured by Western blotting assay. GAPDH was used as a loading control. (b) CD31 levels were normalized to GAPDH and used for statistical analysis. (c, d) The levels of VEGF within the contralateral cerebral cortex of MCAO rats housed in standard cages and enriched environments were also measured by Western blotting assay. (e, f) eNOS levels within the contralateral cerebral cortex of MCAO rats housed in standard cages and enriched environments were measured by Western blotting assay. S: standard cage group; E: enriched environment group. *n* = 6 rats per group.

To examine whether an enriched environment increases blood vessels in the contralateral cerebral cortex of MCAO/r rats, we compared the levels of vascular endothelium in the contralateral cerebral cortices of MCAO/r rats housed in enriched environments and standard cages by analysing the levels of CD31, a specific marker of vascular endothelial cells (Figure 2(a)). There were no differences in the whole contralateral cortical CD31 levels between the enriched environment (*n* = 6 rats) and standard cage (*n* = 6 rats) groups (Figure 2(b)).

We also measured the levels of contralateral cortical vascular endothelial growth factor (VEGF), which plays an important role in angiogenesis and neuroprotection in the brain (Figure 2(c)). There were no differences in VEGF levels within the whole contralateral cerebral cortex between the enriched environment (*n* = 6 rats) and standard cage (*n* = 6 rats) groups (Figure 2(d)). We next measured eNOS levels in the whole contralateral cerebral cortex in MCAO/r rats and did not find obvious differences between the enriched environment (*n* = 6 rats) and standard cage (*n* = 6 rats) groups (Figures 2(e) and 2(f)). These results revealed that a 14-day enriched environment did not enhance angiogenesis in the whole contralateral cerebral cortex in MCAO/r rats.

### 3.3. Enriched Environments Enhanced Angiogenesis Surrounding the Bilateral Cingulate

**Figure 3:**
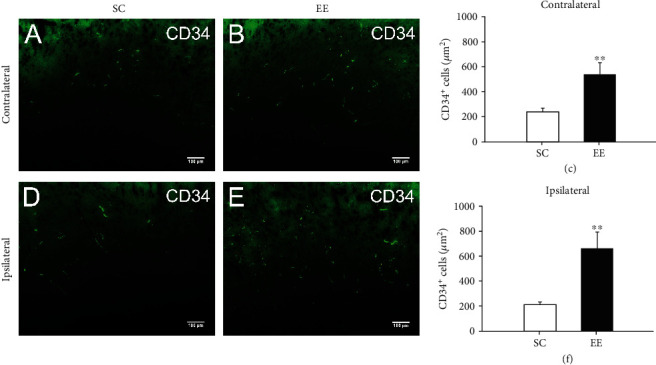
The enriched environment enhanced angiogenesis surrounding the bilateral cingulate. At 14 days after MCAO/r, neovascular endothelial cells were labelled and analysed by immunostaining for CD34. (a, b) Representative images of CD34-positive neovascular endothelial cells surrounding the contralateral cingulate in MCAO/r rats in the standard cage (a) and enriched environment (EE) groups (b). (c) The area of CD34-positive neovascular endothelial cells surrounding the contralateral cingulate in MCAO/r rats in the SE and EE groups was quantified and analysed. *n* = 6 rats per group, ^∗∗^*P* < 0.01, bar = 100*μ*m. (d, e) Representative images of CD34-positive neovascular endothelial cells surrounding the ipsilateral cingulate in MCAO/r rats in the standard cage (d) and enriched environment groups (e). (f) The area of CD34-positive neovascular endothelial cells was quantified and analysed. *n* = 6 rats per group, ^∗∗^*P* < 0.01, bar = 100*μ*m.

Although the above results did not show that an enriched environment increased contralateral cortical angiogenesis in MCAO/r rats, we could not rule out the effect of an enriched environment on blood vessels in the local area. We therefore further examined the effect of enriched environments on angiogenesis by immunostaining CD34-positive endothelial progenitor cells, which are considered to contribute to angiogenesis. We observed a large number of CD34-positive endothelial progenitor cells in or around the cingulate in MCAO/r rats (Figures 3(a) and 3(b)). There were more CD34-positive endothelial progenitor cells within or surrounding the cingulate in MCAO/r rats housed in an enriched environment compared to those housed in a standard environment (*P* = 0.008, *n* = 6, Mann–Whitney *U* test) (Figure 3(c)). Moreover, CD34-positive endothelial progenitor cells surrounding the ipsilateral cingulate in MCAO/r rats housed in an enriched environment were also increased compared with those of rats housed in a standard environment (*P* = 0.001, *n* = 6, Mann–Whitney *U* test) (Figures 3(d)–3(f)).

**Figure 4:**
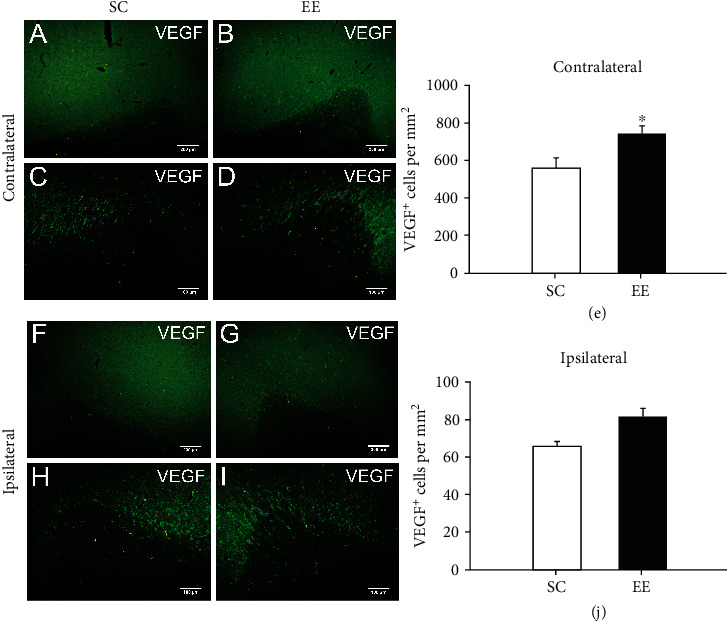
The enriched environment increased VEGF expression surrounding the contralateral cingulate. At 14 days after MCAO/r, VEGF surrounding the bilateral cingulate were measured and analysed by immunostaining. (a–d) Representative images of VEGF-positive cells surrounding the contralateral cingulate in MCAO/r rats in the standard cage ((a) bar = 200*μ*m; (c) bar = 100*μ*m) and enriched environment groups ((b) bar = 200*μ*m; (d) bar = 100*μ*m). (e) The number of VEGF cells surrounding the contralateral cingulate in MCAO/r rats in the SC and EE groups was quantified and analysed. *n* = 6 rats per group,^∗^*P* < 0.05. (f–i) VEGF-positive cells surrounding the ipsilateral cingulate in MCAO/r rats in the standard cage ((f) bar = 200*μ*m; (h) bar = 100*μ*m) and enriched environment ((g) bar = 200*μ*m; (i) bar = 100*μ*m) groups were measured and quantified (j). *n* = 6 rats per group.

Previous studies have shown that an enriched environment enhances cerebral angiogenesis around the ischaemic region in a rat MCAO model by vascular endothelial growth factor (VEGF) and its receptor VEGFR2, and this signalling pathway plays critical roles in angiogenesis and vascular regeneration. We also observed a large number of VEGF-positive cells in or around the cingulate in MCAO/r rats, and these cells significantly increased in and around the contralateral cingulate in MCAO/r rats housed in the enriched environment compared with those of rats housed in the standard environment (*t* = 2.561, *P* = 0.031, *n* = 6, Student's *t*-test) (Figures 4(a)–4(e)). The number of VEGF-positive cells was also increased in and around the ipsilateral cingulate in MCAO/r rats housed in the enriched environment compared with that of rats housed in the standard environment, although there was no significant difference (*n* = 6, Student's *t*-test) (Figures 4(f)–4(j)).

**Figure 5:**
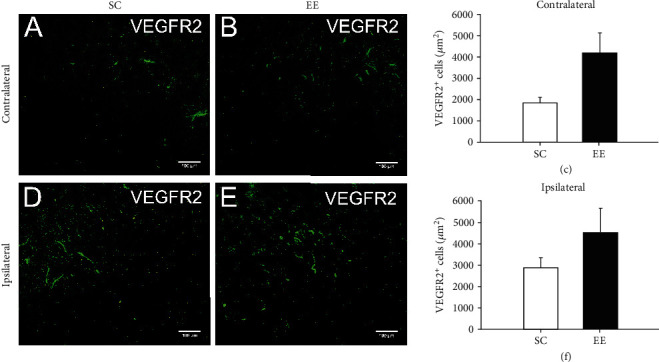
There were more VEGFR2-positive cells in and around the contralateral and ipsilateral cingulate in MCAO/r rats housed in the enriched environment than in rats housed in the standard environment, although there was no significant difference. (a and b) VEGFR2-positive cells surrounding the contralateral cingulate in MCAO/r rats in the standard cage (a) and enriched environment (b) groups were also measured. (c) The area of VEGFR2-positive cells was quantified and analysed. (d–f) VEGFR2-positive cells surrounding the ipsilateral cingulate in MCAO/r rats in the standard cage (d) and enriched environment (e) groups were measured and analysed (f). *n* = 6 rats per group, bar = 100*μ*m.

We further assessed VEGFR2-positive endothelial cells in and around the cingulate in MCAO/r rats housed in an enriched environment and standard environment. There were more VEGFR2-positive cells in and around the contralateral and ipsilateral cingulate in MCAO/r rats housed in the enriched environment than in rats housed in the standard environment, although there was no significant difference (Figures 5(a)–5(f)).

**Figure 6:**
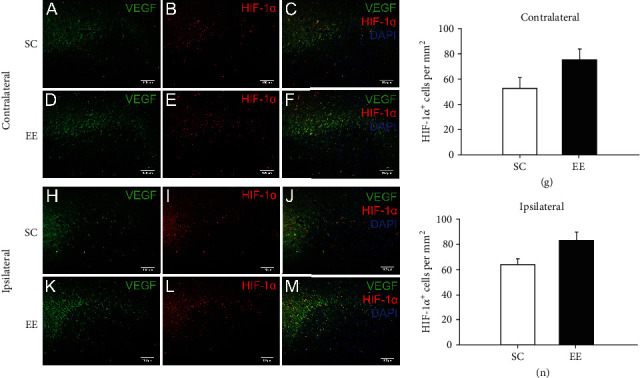
VEGF-positive cells were colocalised with HIF-1*α* surrounding the bilateral cingulate. Both VEGF and HIF-1*α* were immunostained. The HIF-1*α*-positive cells surrounding the bilateral cingulate were measured at 14 days after MCAO/r. (a, d) Representative images of VEGF-positive cells surrounding the contralateral cingulate in MCAO/r rats in the standard cage (a) and enriched environment groups (d). (b, e) Representative images of HIF-1*α*-positive cells surrounding the contralateral cingulate in MCAO/r rats in the standard cage (b) and enriched environment groups (e). (c, f) Almost all VEGF-positive cells surrounding the contralateral cingulate in MCAO/r rats in the standard cage (c) and enriched environment groups (f) were colabelled with HIF-1*α*. (h, k) Representative images of VEGF-positive cells surrounding the ipsilateral cingulate in MCAO/r rats in the standard cage (h) and enriched environment groups (k). (i, l) Representative images of HIF-1*α*-positive cells surrounding the ipsilateral cingulate in MCAO/r rats in the standard cage (i) and enriched environment groups (l). (j, m) Almost all VEGF-positive cells surrounding the ipsilateral cingulate in MCAO/r rats in the standard cage (j) and enriched environment groups (m) were colabelled with HIF-1*α*. (g, n) The number of HIF-1*α* cells surrounding the contralateral (g) and ipsilateral (n) cingulate in MCAO/r rats in the SC and EE groups was quantified and analysed. *n* = 6 rats per group, bar = 100*μ*m.

Moreover, we measured the predominant factors that upregulate VEGF expression. Hypoxia-inducible factor-1*α* (HIF-1*α*) is recognized as a regulator of hypoxic signalling. By activating gene transcription, HIF-1*α* could upregulate VEGF expression, thus increasing angiogenesis. By colabelling HIF-1*α* and VEGF, as shown in Figure 6, almost all VEGF-positive cells in and around the bilateral cingulate in MCAO/r rats expressed HIF-1*α* (Figures 6(a)–6(f), 6(h)–6(m)). Furthermore, the expression levels of HIF-1*α* were increased in and around the contralateral and ipsilateral cingulate in MCAO/r rats housed in an enriched environment compared with those housed in a standard environment, although there was no significant difference (Figures 6(g) and 6(n)). Overall, these results suggested that the enriched environment increased the expression of VEGF in and around the contralateral and ipsilateral cingulate in MCAO/r rats by upregulating HIF-1*α*, which enhanced VEGF-induced angiogenesis within this brain region.

### 3.4. Enriched Environments Increased Endothelial eNOS Expression Surrounding the Cingulate

**Figure 7:**
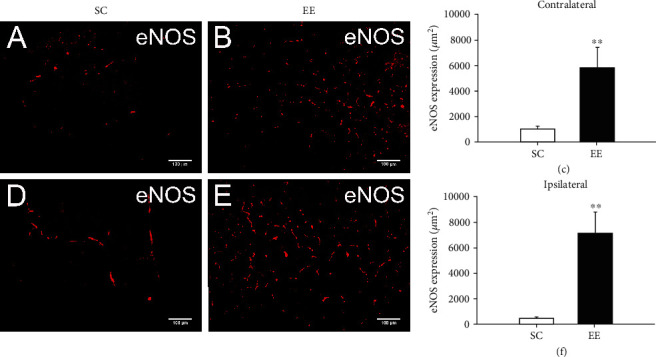
The enriched environment increased eNOS generation surrounding the bilateral cingulate. The expression of eNOS in endothelial cells surrounding the bilateral cingulate was analysed by immunostaining at 14 days after MCAO/r. (a, b) Representative images of eNOS-positive endothelial cells surrounding the contralateral cingulate in MCAO/r rats in the standard cage (a) and enriched environment groups (b). (c) The area of eNOS-positive endothelial cells surrounding the contralateral cingulate in MCAO/r rats in the SC and EE groups was used for statistical analysis. *n* = 6 rats per group, ^∗∗^*P* < 0.01, bar = 100*μ*m. (d–f) eNOS-positive cells surrounding the ipsilateral cingulate in MCAO/r rats in the standard cage (d) and enriched environment groups (e) were also analysed (f). *n* = 6 rats per group, ^∗∗^*P* < 0.01, bar = 100*μ*m.

Endothelial nitric oxide synthase (eNOS) is a critical downstream molecule of VEGF/VEGFR2 signalling and plays a predominant role in VEGF-induced angiogenesis and vascular permeability. Therefore, we further examined the influence of the enriched environment on the expression of eNOS in and around the bilateral cingulate in MCAO/r rats. The enriched environment significantly increased the expression of eNOS in and around the contralateral cingulate in MCAO/r rats (*t* = 3.171, *P* = 0.007, *n* = 6, Student's *t*-test) (Figures 7(a)–7(c)), as well as in and around the ipsilateral cingulate (*t* = 3.690, *P* = 0.003, *n* = 6, Student's *t*-test), in comparison with that of rats housed in the standard environment (Figures 7(d)–7(f)).

**Figure 8:**
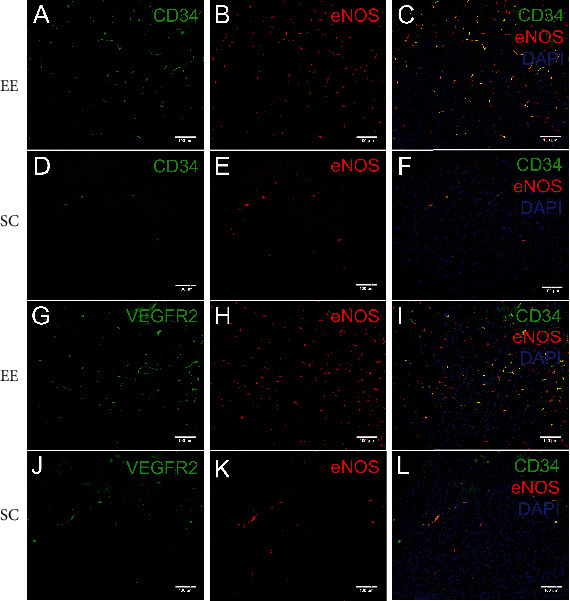
Almost all CD34-positive cells surrounding the contralateral cingulate in MCAO/r rats were colabelled with eNOS, and almost all eNOS-positive cells surrounding the contralateral cingulate in MCAO/r rats were colabelled with VEGFR2. (a–f) Almost all CD34-positive cells surrounding the contralateral cingulate in MCAO/r rats were colabelled with eNOS. (a, d) Representative images of CD34-positive endothelial cells surrounding the contralateral cingulate in MCAO/r rats in the enriched environment (a) and standard cage groups (d). (b, e) Representative images of eNOS-positive endothelial cells surrounding the contralateral cingulate in MCAO/r rats in the enriched environment (b) and standard cage groups (e). (c, f) Almost all CD34-positive cells surrounding the contralateral cingulate in MCAO/r rats in the enriched environment (c) and standard cage groups (f) were colabelled with HIF-1*α*. (g–l) Almost all eNOS-positive cells surrounding the contralateral cingulate in MCAO/r rats were colabelled with VEGFR2. (g, j) Representative images of VEGFR2-positive endothelial cells surrounding the contralateral cingulate in MCAO/r rats in the enriched environment (g) and standard cage groups (j). (h, k) Representative images of eNOS-positive endothelial cells surrounding the contralateral cingulate in MCAO/r rats in the enriched environment (h) and standard cage groups (k). (i, l) Almost all eNOS-positive cells surrounding the contralateral cingulate in MCAO/r rats in the enriched environment (i) and standard cage groups (l) were colabelled with VEGFR2. Bar = 100*μ*m.

We next examined the involvement of eNOS in the enriched environment-mediated improvement in angiogenesis by the VEGF/VEGFR2 signalling pathway. Almost all CD34-positive endothelial progenitor cells were colocalized with eNOS in and around the cingulate in MCAO/r rats (Figures 8(a)–8(f)), which revealed the essential role of eNOS in increasing angiogenesis in response to the enriched environment. We also examined the expression of eNOS in VEGFR2-positive endothelial cells and found that almost all eNOS-positive cells surrounding the contralateral cingulate in MCAO/r rats were colabelled with VEGFR2. However, a small number of VEGFR2-positive endothelial cells may not be eNOS-positive (Figures 8(g)–8(l)). These results further confirmed the involvement of eNOS in VEGF/VEGFR2 signalling-induced angiogenesis, which was enhanced in and around the bilateral cingulate in MCAO/r rats housed in the enriched environment.

## 4. Discussion

Enriched environmental stimulation stimulates the whole brain. The brain region contralateral to the infarction, with complete structure and function, may exhibit improved responses to environmental stimulation. Previous animal experiments have shown that after cerebral infarction, the contralateral hemisphere has obvious nerve regeneration and glial cell proliferation, which can compensate for the partial functions of the injured side and promote neurological functions [1, 22, 23].

In our previous study, EEs increased the expression levels of VEGF and its receptor after ischaemia-reperfusion injury in the ipsilateral hemisphere and improved neural functional outcomes [20]. In this study, we measured and compared angiogenesis in the contralateral hemisphere and found that compared with standard cage feeding, 14-day enriched environmental feeding did not increase the vascular density in the whole infarcted contralateral hemisphere cortex. For Western blotting assay, we collected the whole contralateral cerebral cortex, homogenised it to extract the proteins, and discarded the subcortical part. Therefore, it is possible that the total amount of cortical protein remained unchanged, but the local distribution changed. Western blotting assay also could not reflect the protein changes in the subcortical structure. VEGF can also be expressed in subcortical structures far from the infarct (such as the corpus callosum and hippocampus) and cause angiogenesis, which may mitigate penumbra and white matter injury following ischaemic stroke [24, 25]. We assessed angiogenesis in subcortical structures or local areas of the contralateral cerebral cortex.

We used immunofluorescence to identify angiogenesis in the infarcted contralateral hemisphere, further observed and compared the different cerebral regions in the infarcted contralateral hemisphere, and found that there were cells with high expression of VEGF in and around the cingulate belt. Through comparison, it was found that enriched environmental intervention could increase the number of VEGF-positive cells in this region. In addition, most VEGF-positive cells also expressed HIF-1*α*, and the number of HIF-1*α*-positive cells increased in the enriched environment group compared with the standard cage group, although there was no statistically significant difference. This finding suggests that the EE may increase VEGF secretion by promoting the expression of the transcription factor HIF-1*α* [26, 27]. However, the mechanism by which the enriched environment promotes the expression of VEGF in and around the cingulate needs further study.

The main function of VEGF is to promote angiogenesis [13]. Our results also showed that the environment significantly increased CD34-positive endothelial progenitor cells around both cingulate sites in MCAO model rats [28]. VEGFR2 expression was increased in vascular endothelial cells in this area at the same time. Further studies showed that CD34- and VEGFR2-positive cells can also express eNOS, and the enriched environment significantly enhanced the expression of eNOS in this region. eNOS is an important factor associated with VEGF in the regulation of angiogenesis and vascular function that can regulate angiogenesis and microvascular function by synthesizing nitric oxide (NO) [29, 30]. This finding suggests that the enriched environment may enhance nearby angiogenesis by promoting the expression of VEGF in the cingulate cortex and regulating the microcirculation in the cingulate and nearby regions [28, 31]. In addition, some studies have shown that VEGF enhances the tolerance of nerve cells to hypoxia during stroke and has a direct neuroprotective effect [32, 33]. It has also been reported that after brain injury, VEGF can promote nerve regeneration, improve synaptic plasticity, and promote nerve function recovery [27, 34]. This finding suggests that the EE has neuroprotective effects by improving local microcirculation. The EE may promote neuronal regeneration and enhance the function of the contralateral cingulate gyrus after stroke by promoting angiogenesis, which may be significant for the recovery of neurological function after stroke.

The lack of functional verification results has become a limitation of this study. A more functional study may help us to know more about how the VEGF-eNOS pathway to bilateral cingulate cortices affects the recovery efficacy of MCAO/r.

It has been confirmed that after partial brain injury, the contralateral hemisphere can compensate for the partial function of the injured side and promote the recovery of neurological function [35–37]. The present study showed that EEs may enhance function around the cingulate gyrus. Studies have demonstrated that cingulate neurons can project into the hippocampal and the parahippocampal gyrus and participate in the information integration processes of learning and memory [38]. In addition, some cingulate cortex neurons can project into the M1 region (primary motor cortex) and participate in the regulation of head, face, and forelimb movements [39]. In our previous study, EEs promoted cognitive function and improved the recovery of coordination and the integration of motor movements [40–42]. This finding suggests that EEs may promote the recovery of movement and participate in the learning and remodelling of poststroke movement by activating the VEGF-eNOS pathway in and around the cingulum, promoting the function of the cingulate cortex [43]. The cingulate, mainly the anterior cingulate cortex, monitors the consequences of actions and mediates subsequent changes in behaviour [44]. Stroke leads to poststroke depression or anxiety, which may be related to dysfunction in the cingulate gyrus [45]. EEs can reduce the aversion of stroke patients to rehabilitation treatment and promote patient enthusiasm, which may be related to promoting behaviour monitoring and reward in the cingulate gyrus [46, 47]. This finding suggests that enriched environmental therapy can not only promote the recovery of neuromotor function after stroke but also prevent and improve the patient poststroke emotional abnormalities by promoting the function of the cingulate gyrus [48]. EEs may promote recovery by promoting enthusiasm for treatment. This also suggests that in the process of rehabilitation treatment of stroke patients, especially patients with low mood, apathy, and negative emotions, EE stimulation can be used as an important auxiliary treatment for patients [49].

## Data Availability

The data used to support the findings of this study are available from the corresponding author upon request.
